# Efficacy and safety of the second in-hospital dose of tranexamic acid after receiving the prehospital dose: double-blind randomized controlled clinical trial in a level 1 trauma center

**DOI:** 10.1007/s00068-021-01848-0

**Published:** 2021-12-15

**Authors:** Ayman El-Menyar, Khalid Ahmed, Suhail Hakim, Ahad Kanbar, Saji Mathradikkal, Tariq Siddiqui, Hisham Jogol, Basil Younis, Ibrahim Taha, Ismail Mahmood, Ahmed Ajaj, Sajid Atique, Abubaker Alaieb, Ahmed Abdel-Aziz Bahey, Mohammad Asim, Guillaume Alinier, Nicholas R. Castle, Ahammed Mekkodathil, Sandro Rizoli, Hassan Al-Thani

**Affiliations:** 1 Trauma & Vascular Surgery, Clinical Research, Hamad General Hospital, Hamad Medical Corporation (HMC), P.O Box 3050, Doha, Qatar; 2 Clinical Medicine, Weill Cornell Medical College, Doha, Qatar; 3 Department of Surgery, Trauma Surgery, Hamad General Hospital, HMC, Doha, Qatar; 4 Trauma Surgery, Clinical Pharmacy, Hamad General Hospital, HMC, Doha, Qatar; 5 Hamad Medical Corporation Ambulance Service, Medical City, HMC, Doha, Qatar; 6 Weill Cornell Medicine-Qatar, Doha, Qatar; 7 School of Health and Social Work, Paramedic Division, University of Hertfordshire, Hatfield, UK; 8 Faculty of Health and Life Sciences, Northumbria University, Newcastle upon Tyne, UK

**Keywords:** Trauma, Tranexamic acid, Bleeding, Randomized controlled trial, Prehospital

## Abstract

**Background:**

Prehospital administration of tranexamic acid (TXA) to injured patients is increasing worldwide. However, optimal TXA dose and need of a second infusion on hospital arrival remain undetermined. We investigated the efficacy and safety of the second in-hospital dose of TXA in injured patients receiving 1 g of TXA in the prehospital setting. We hypothesized that a second in-hospital dose of TXA improves survival of trauma patients.

**Methods:**

A prospective, double-blind, placebo-controlled randomized, clinical trial included adult trauma patients receiving 1 g of TXA in the prehospital settings. Patients were then blindly randomized to Group I (second 1-g TXA) and Group II (placebo) on hospital arrival. The primary outcome was 24-h (early) and 28-day (late) mortality. Secondary outcomes were thromboembolic events, blood transfusions, hospital length of stay (HLOS) and organs failure (MOF).

**Results:**

A total of 220 patients were enrolled, 110 in each group. The TXA and placebo groups had a similar early [OR 1.000 (0.062–16.192); *p* = 0.47] and late mortality [OR 0.476 (95% CI 0.157–1.442), *p* = 0.18].The cause of death (*n* = 15) was traumatic brain injury (TBI) in 12 patients and MOF in 3 patients. The need for blood transfusions in the first 24 h, number of transfused blood units, HLOS, thromboembolic events and multiorgan failure were comparable in the TXA and placebo groups. In seriously injured patients (injury severity score > 24), the MTP activation was higher in the placebo group (31.3% vs 11.10%, *p* = 0.13), whereas pulmonary embolism (6.9% vs 2.9%, *p* = 0.44) and late mortality (27.6% vs 14.3%, *p* = 0.17) were higher in the TXA group but did not reach statistical significance.

**Conclusion:**

The second TXA dose did not change the mortality rate, need for blood transfusion, thromboembolic complications, organ failure and HLOS compared to a single prehospital dose and thus its routine administration should be revisited in larger and multicenter studies.

**Trial registration:**

ClinicalTrials.gov Identifier: NCT03846973.

## Introduction

Traumatic injuries represent 10% of the disease burden worldwide and particularly trauma-induced hemorrhage which is the most preventable cause of mortality among injured patients [1–4]. Around one-fourth of trauma patients are susceptible to develop acute coagulopathy and those with uncontrolled hemorrhage are at increased risk of hemorrhagic shock and mortality [4–8]. Therefore, early management of acute traumatic coagulopathy and hemorrhagic shock improves outcomes [5, 9]. Following a major hemorrhage, tranexamic acid (TXA), a synthetic version of the amino acid lysine, inhibits the premature breakdown of blood clots [10, 11]. Evidence suggests that administration of TXA immediately or within 3 h of injury is associated with survival benefit in trauma patients [12–19]. Several meta-analyses have been done to explore the risk and benefits of TXA in trauma [20–23]. These meta-analyses, however, contain few studies on prehospital administration of TXA [1, 20], whereas most were conducted in the hospital settings [21, 23–26]. Evidence indicates that TXA reduces death secondary to bleeding by as much as 32% if given within 1 h of injury, but may increase death if given after 3 h [27]. Since the publication of CRASH-2, most of hospitals have adopted TXA administration as 1 g bolus, followed by 1 g infusion. Apart from the data from the cardiac surgery on the TXA dosage, the optimal TXA timing, dosage, and administration frequency to injured patients are not well studied and remain undetermined [28]. Although TXA has a half-life of 2.3 h in adult patients [29], one study showed that in one-fifth of cases, a single dose of TXA given en route to the hospital did not attain adequate plasma level measured at hospital admission [27]. Moreover, TXA administration may increase the risk of thromboembolic complications, particularly when higher or repeated doses are used as shown in the MATTERS and HALT-IT trials [21, 30, 31]. Moreover, there are few studies on the efficacy and safety of TXA en route to the hospital or at the scene [20, 32–35].

Despite the limitations, as more evidence is published, prehospital TXA administration has gradually been adopted by many international emergency medical services (EMS), including in Qatar. In few countries, prehospital TXA is considered standard of care for injured adult patients at risk of hemorrhage and more recently head injury [7, 16–18, 32, 36, 37]. However, a recent report found a large geographical variance in the use of TXA in trauma, particularly in Asia and Africa [33]. The present randomized controlled trial (RCT) aimed to evaluate the safety and efficacy of the second in-hospital dose of TXA versus placebo on mortality and complications in adult trauma patients receiving the first TXA dose prehospitally. We hypothesized that a second dose of TXA on hospital arrival improves survival, but at the expense of more thromboembolic complications.

## Methods

### Study design and population

We conducted a prospective, double-blind, randomized, placebo-controlled clinical trial on the efficacy and safety of the second in-hospital dose of TXA on mortality, thromboembolic complications, blood transfusions, hospital stay and organ failure in adult trauma patients receiving the first 1 g TXA dose prior to hospital arrival. The clinical trial was conducted at the sole tertiary level 1 trauma center at Hamad General Hospital (HGH) in Qatar, between December 2018 and January 2021. Adult injured patients receiving an initial bolus TXA dose of 1 g in the prehospital setting were eligible for inclusion. The prehospital dose of TXA was administered by the critical care paramedics (CCP) as a standard of care treatment for risk of bleeding as per the CRASH-2 criteria [15]. The study excluded patients older than 90 or younger than 18 years, those on whom intravenous access could not be established, documented cervical cord injury with motor deficit, prisoners, known pregnancy, traumatic cardiac arrest for over 5 min unsuccessful cardio-pulmonary resuscitation, penetrating cranial injury, traumatic brain injury (TBI) with brain matter exposed, isolated drowning, hanging victims, and severe renal failure patients. Written informed consents were obtained from the subjects or next of kin (proxy) if subjects were incapacitated. If proxy was unavailable, then deferred consent was used as per institutional guidelines. For those who regained consciousness after admission, written informed consent was obtained for continuation in the study and utilization of anonymously collected data with secured confidentiality. The Institutional Review Board of Hamad Medical Corporation approved the clinical trial (IRB# 16417/16) which was registered in the ClinicalTrials.gov (Identifier: NCT03846973) and follows Consolidated Standards of Reporting Trials (CONSORT) checklist.

### Emergency medical services

In Qatar, most trauma cases are blunt (> 85%), primarily due to road traffic injuries and fall from height [38]. All severely injured patients in Qatar are transported and treated at no cost at the sole level 1 trauma center at HGH. The Hamad Medical Corporation Ambulance Service (HMCAS) is the national ambulance service of Qatar, responding up to 270,000 emergency calls per year, of which around 19% are trauma. All emergency calls are centrally managed by the National Command Centre and monitored against established national response time targets.

HMCAS operates a hub-and-spoke model to optimize response, ensuring that a minimum of 75% of emergency calls from the urban setting are reached by an ambulance within 10 min and 20 min in rural areas [39]. The EMS vehicles are equipped with the latest digital health technologies (i.e., mobile data terminals with global positioning systems, electronic patient care record tablets, traffic light management systems, telemetry features and asset radio frequency identification tags) and medical equipment allowing the trained crews to respond to all types of emergency situations [40].

HMCAS operates a tiered response system with the primary responsibility based on a dual-staffed paramedic ambulance supported by CCPs, responding on rapid response vehicles or a helicopter platform [39]. Compared to ambulance paramedics, CCPs have a broader scope of practice including administration of TXA, anesthesia drugs, and other life-saving techniques, except blood transfusions that are not available for prehospital use [41].

### Prehospital TXA administration

Prehospital TXA was adopted by the CCP scope of practice in Qatar since June 2016. Based on data from January 2019 to March 2021, prehospital TXA administration occurs on an average 28 times per month in Qatar, with a noticeable increase over time, from about 16 to 37 doses per month. For the current trial, potential subjects were identified by the CCP during handover to the trauma team, with documented evidence of administration of the first dose of TXA as the standard of prehospital care. TXA was administrated by the CCPs according to the CRASH-2 criteria for patients suspected or confirmed to have bleeding using the following parameters: systolic blood pressure (SBP) ≤ 90 mmHg, heart rate (HR) ≥ 110 beats per min, or both at any time,

### Randomization and masking

After arrival at the hospital and obtaining eligibility confirmation and consent, patients were blindly assigned to either the treatment or placebo arms as per the randomization scheme. Randomization (1:1) was balanced with an allocation sequence based on a block size of eight, generated with a computer random number generator. Subjects were considered enrolled in the study after opening the opaque envelope of the randomization series kept in the trauma room. The envelopes contained a randomization code, a wristband for the patient, and a label for the study number with randomization code to be placed in the subject’s medical record.

Both participants and study staff (investigators and trial staff) were masked to treatment allocation. Blinding was done through the pharmacy department responsible for preparing and supplying the treatment packs for the study with an identical ampoule of TXA and placebo (0.9% saline).

### Intervention

The clinical trial had two arms and recruited patients were randomly assigned to either a second 1-g dose of TXA or placebo (normal saline) on hospital admission. After treatment randomization, the treating physician administered intravenous infusion of 1 g TXA or placebo, over 8 h. All patients, regardless of group assignment, were managed equally and according to established standards of care.

Ten ml blood specimens were drawn (5 ml before and 5 ml after the infusion) for assessment of the coagulation profile including: international normalized ratio (INR), prothrombin time (PT), activated partial thromboplastin time (aPTT), fibrinogen, and D-dimer. Data were prospectively collected for demographics (age, gender, nationality), injury characteristics, mechanism of injury, vital signs at the scene and after randomization, time from injury to randomization, shock index (prehospital and at ED), laboratory findings on initial presentation to ED and post-randomization, AIS, ISS, blood transfusion details (overall and within 24 h post-trauma), surgical interventions, mechanical ventilation, ICU and hospital stay, in-hospital complications and mortality. CT pulmonary angiogram and ultrasound Doppler were used for patients suspected to have thromboembolic complications. Massive transfusion was defined as receiving ≥ 10 blood units during the first 24 h. Shock index was defined as HR divided by simultaneous SBP [42]. An ISS of 1–8 is considered minor, 9–15 moderate, 16–24 severe, and 25 and higher critical injury [43].

### Outcome measures

The primary outcome measure was mortality at 24 h (early) and 28 days (late) post-injury. Secondary outcome measures were in-hospital thromboembolic complications (pulmonary embolism—PE and deep vein thrombosis), multiorgan failure (MOF), blood transfusions, massive transfusion protocol activation, and hospital length of stay.

### Follow-up

The study primary outcomes were assessed at 4 weeks post-injury. In case of early discharge from the hospital, the subjects were contacted through telephone. All subjects in the trial were followed up for 28 days irrespective of whether they received the study treatment or withdrawn.

### Statistical analysis

The sample size was calculated using a mortality estimate of 16% according to the CRASH-2 trial [15, 24], a two-sided alpha = 0.05 with 85% power to detect a difference of 12% points in the 28-day mortality (16% vs 4%) between the two study groups (TXA versus placebo). The required sample size was 220 trauma patients (110 receiving the second dose of TXA and 110 receiving placebo). We assumed that receiving two doses of TXA will have robust effect on the outcome.

Data were reported as proportion, mean (95% confidence intervals), median, and interquartile range (IQR), when applicable. Clinical characteristics, injury severity, and outcomes were first compared among the two study groups (Group I: prehospital TXA + in-hospital second dose of TXA vs Group II: prehospital TXA + in-hospital Placebo). The study groups were compared using *χ*^2^ test for categorical variables and Student *T* test for comparison of continuous variables. For cell values less than 5, Yates corrected Chi square was used. For skewed continuous data, non-parametric Mann–Whitney *U* test or Kruskal–Wallis test was performed as appropriate. Kaplan–Meier survival curve is used to analyze time to event and to compare the groups of subjects (Groups-I and -II). A significant difference was considered when the two-tailed p value was less than 0.05. We calculated odd ratios (OR) and 95% confidence interval (CI) for each binary outcome and two-sided p values for statistical significance. All analyses were undertaken on an intention-to-treat basis. Data analysis was carried out using the Statistical Package for Social Sciences version 21 (SPSS Inc. Chicago, Illinois, US).

## Results

**Fig. 1:**
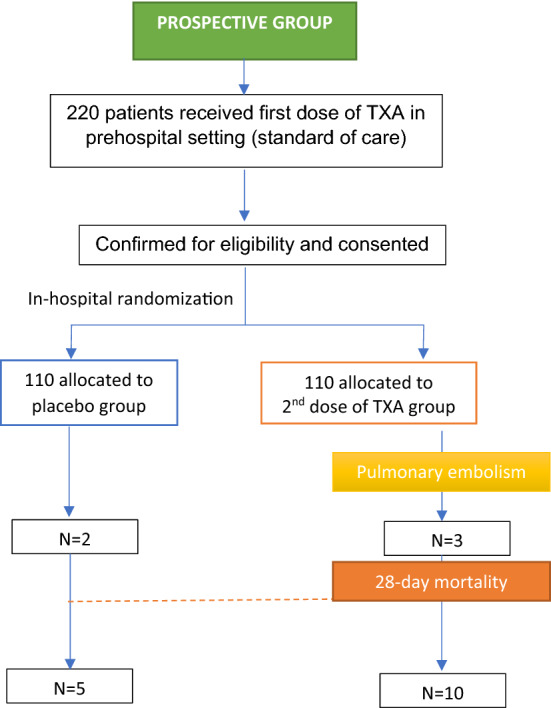
Study flow diagram for the hospital tranexamic acid (TXA) and control group. *TXA* tranexamic acid; *ISS* injury severity score; *AIS* abbreviated injury score; SBP: systolic blood pressure

A total of 220 patients were deemed eligible for enrollment, with 110 randomized to the TXA Group I and 110 to the placebo Group II. All patients enrolled received the full intervention, TXA or placebo. Figure 1 shows the flowchart for the study design.

Table 1 displays patient characteristics by treatment group (TXA vs. placebo). The two groups were balanced for baseline demographic characteristics such as age, gender, or nationality. The mean age of the subjects in both groups was nearly 34 years and most were males (94% TXA vs. 98% placebo). Blunt trauma (86% vs. 85%) was the most mechanism of injury (13% vs. 11%), mostly resulting from road traffic crashes (65% vs. 63%) and fall from height (15% vs. 20%) with no significant difference (*p* > 0.05).

**Table 1:** Patient characteristics by treatment group

Characteristics	Group I: PHTx + H TXA (*n* = 110)	Group II: PHTx + H placebo (*n* = 110)	*p* value
Age (years)	34.1 (32.6–36.8)	33.9 (31.9–35.8)	0.55
Males	103 (93.6%)	108 (98.1%)	0.08
Type of injury			
Blunt	94 (85.5%)	93 (84.5%)	0.50
Penetrating	14 (12.7%)	12 (10.9%)	0.83
Both	02 (1.8%)	05 (4.5%)	0.44
Mechanism of injury			
Road traffic accident	71 (64.5%)	69 (62.7%)	0.89
Fall from height	16 (14.5%)	22 (20%)	0.37
Assault	11 (10%)	11 (10%)	0.82
Others	12 (10.9%)	08 (7.2%)	0.48
Prehospital SBP (mmHg)	117.4 (111.2–123.6)	118.8 (113.9–123.7)	0.73
Prehospital shock index	0.92 (0.85–0.98)	0.90 (0.84–0.96)	0.62
SBP (mmHg) after randomization	120.2 (115.7–124.7)	118.6 (114.1–123.2)	0.63
Prehospital RR (bpm)	23.3 (21.8–24.7)	24.7 (22.9–26.4)	0.23
RR after randomization (bpm)	20.7 (19.7–21.6)	21.3 (20.2–22.5)	0.36
Prehospital HR (BPM)	100.8 (95.9–105.6)	101.2 (96.6–105.6)	0.91
HR after randomization (bpm)	98.1 (94.2–102.0)	98.3 (93.8–102.8)	0.94
In-hospital SBP ≤ 90	11.8%	14.5%	0.55
Shock index at ED	0.86 (0.80–0.92)	0.89 (0.82–0.99)	0.49
Initial GCS at presentation	11.2 (10.3–12.1)	11.8 (10.9–12.7)	0.29
GCS after randomization	10.5 (9.6–11.5)	10.9 (9.9–11.9)	0.58
On-hospital admission			
Hemoglobin (g/dl)	13.04 (12.7–13.4)	12.9 (12.5–13.3)	0.61
Platelets (10^3^/µL)	251.1 (236.3–265.9)	254.1 (241.9–266.4)	0.75
INR (mg/dl)	1.18 (1.14–1.23)	1.2 (1.15–1.23)	0.91
PT (s)	12.8 (12.3–13.2)	12.7 (12.4–13.2)	0.93
APTT (s)	26.9 (25.3–28.7)	27.9 (25.7–30.1)	0.52
Blood lactate (mmol/L)	3.2 (2.9–3.7)	3.3 (2.9–3.7)	0.93
Base deficit	− 5.73 (− 6.53 to 4.93)	− 5.4 (− 6.27 to 4.67)	0.64
Fibrinogen (gm/L)	2.8 (1.3–4.3)	2.03 (1.9–2.2)	0.29
d-dimer (mg/L)	18.6 (13.8–23.4)	21.8 (17.3–26.2)	0.33

Data are presented as number (%) for categorical variables and mean, 95% CI for continuous variables

*TXA* tranexamic acid, *PHTx* prehospital TXA, *H* hospital

The two groups were comparable for vital signs, hematological parameters (Hb, serum lactate, base deficit, INR, fibrinogen, and D-dimer), injury severity and Glasgow Coma Scale (GCS). Shock index (SI) was high in both study arms, it was > 0.90 prehospitally and > 0.8 at the ED. Prehospital SBP ≤ 90 mmHg was observed in 25.5% and 16.4% in Group I and II, respectively, *p* = 0.09.

Table 2 shows injury characteristics and blood transfusion by treatment group. No significant differences were observed with respect to SI, associated injuries, abbreviated injury scores for various anatomical regions and ISS. Table 3 shows the time intervals in both groups. The mean times from injury to prehospital TXA, from injury to ED admission and from ED admission to the second dose were comparable in the study groups. The mean time from injury to randomization was 165 min for the TXA group and 172 min for the placebo group (*p* = 0.48).

**Table 2:** Injury characteristics and blood transfusion by treatment group

	Group I: PHTx + H TXA	Group II: PHTx + H placebo	*p* value
Associated injuries			
Head	41 (37.3%)	45 (40.9%)	0.58
Chest	58 (52.7%)	66 (60.0%)	0.27
Abdomen	32 (29.1%)	39 (35.5%)	0.31
Pelvis	33 (30.0%)	29 (26.4%)	0.54
Head AIS	3.3 (2.3–3.6)	3.5 (3.2–3.8)	0.43
Chest AIS	2.8 (2.6–3.01)	2.7 (2.5–2.9)	0.74
Abdomen AIS	2.7 (2.4–3.1)	2.6 (2.3–2.9)	0.49
Pelvis AIS	2.2 (2.06–2.4)	2.4 (2.5–2.7)	0.10
Injury severity score (ISS)	19.2 (17.2–21.1)	19.6 (17.9–21.9)	0.60
Number of blood units transfused	7.5 (5.4–9.6)	6.2 (4.4–8.1)	0.97
No. of blood units given (≤ 24 h)	5.1 (3.1–7.0)	1.92 (1.14–2.7)	0.13
PRBCs transfusion (≤ 24 h)	1.06 (0.5–1.6) (*n* = 28)	1.27 (0.8–1.7) (*n* = 33)	0.55
Plasma transfusion (≤ 24 h)	0.73 (0.3–1.1) (*n* = 10)	0.6 (0.3–0.9) (*n* = 15)	0.60
Platelet transfusion (≤ 24 h)	0.13 (0.02–0.3) (*n* = 2)	0.05 (0.05–0.2) (*n* = 1)	0.39
Surgical intervention	1 (0.9%)	3 (2.7%)	0.25
Mechanical ventilation	54 (49.09%)	48 (43.6%)	0.49
Ventilatory days	9.0 (4.0–13.9)	6.8(4.1–9.5)	0.45
ICU admission	72 (65.4%)	79 (71.8%)	0.38
ICU length of stay	9.9 (7.1–12.6)	8.2 (6.1–10.2)	0.32
Hospital length of stay	21.4 (17.4–25.4)	20.5 (16.2–24.8)	0.77

Data are presented as number (%) for categorical variables and mean, 95% CI for continuous variables

*TXA* tranexamic acid, *PHTx* prehospital TXA, *H* hospital

**Table 3:** Time intervals for the study groups

	Group I: PHTx + H TXA (*n* = 110)	Group II: PHTx + H Placebo (*n* = 110)	*p* value
Time from injury to prehospital TXA (min)	53.4 (47.1–59.6)	54.2 (47.8–60.6)	0.85
Time from injury to ED admission (min)	86.4 (79.8–92.9)	86.8 (78.2–95.5)	0.94
Time from injury to randomization (min)^a^	165.2 (148.7–181.6)	172.4 (160.1–184.7)	0.48
Time from ED admission to second dose^b^ (min)	196.4 (171.4–221.3)	175.7 (158.3–193.1)	0.18
Time from injury to second dose^b^ (min)	282.7 (256.1–309.4)	262.5 (242.8–282.3)	0.23
Number of patients recruited after 3 h (%)^b^	33 (30%)	44(40%)	0.12

Data are presented as number (%) for categorical variables and mean, 95% CI for continuous variables

*TXA* tranexamic acid, *PHTx* prehospital TXA, *H* hospital

^a^Time needed for re-assessment, imaging and consenting to participate, and envelop opening

^b^TXA or placebo

**Fig. 2:**
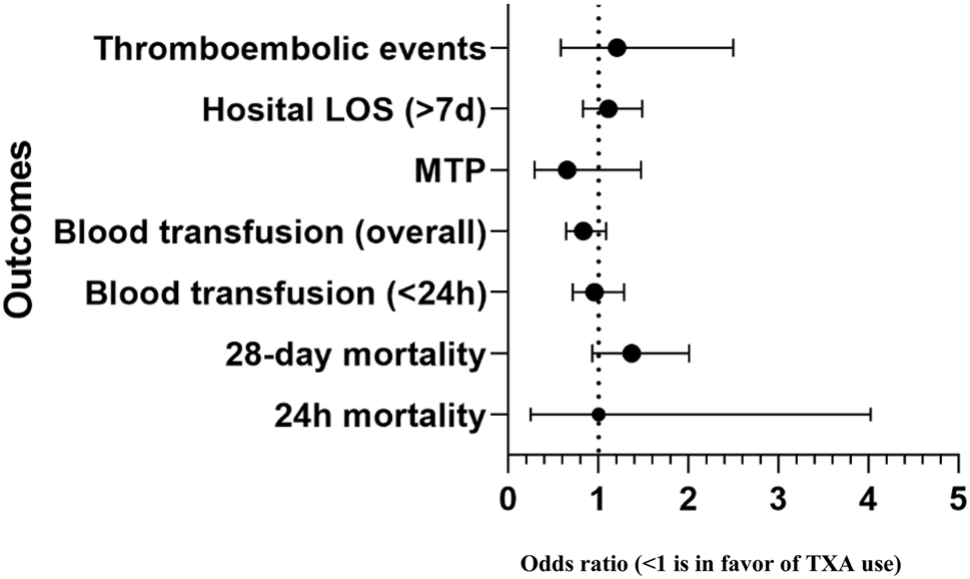
Comparison of outcomes by treatment groups (second dose of TXA vs placebo)

The primary outcome was available for all patients. At 24 h post-injury, one death (0.9%) occurred in the tranexamic acid intervention group and one death (0.9%) in the placebo group (Table 4 and Fig. 2). At 28 days post-injury, ten patients (9.1%) died in the TXA Group I versus five (4.5%) in the placebo Group II. Despite the twofold difference in the number of late deaths, it did not reach statistical significance in both early and late mortality (*p* > 0.05). Concerning the cause of death, eight of the ten deaths in the TXA group died due to severe head injuries and two died with MOF, whereas, in the placebo group, four out five deaths were due to severe TBI and one had MOF.

**Table 4:** Comparison of outcomes by treatment group

	Group I: PHTx + H TXA	Group II: PHTx + H placebo	Risk ratio (95% CI)	*p* value
Primary outcome				
24 h mortality	1 (0.9%)	1 (0.9%)	1.000 (0.248–4.024)	0.47
28-day mortality	10 (9.1%)	5 (4.5%)	1.367 (0.930–2.007)	0.18
Secondary outcomes				
Blood transfusion (< 24 h)	32 (29.1%)	34 (30.9%)	0.957 (0.713–1.284)	0.76
Blood transfusion overall	51 (46.4%)	61 (55.5%)	0.833 (0.639–1.087)	0.17
Massive transfusion protocol activation	4 (12.5%)	8 (23.5%)	0.654 (0.290–1.472)	0.25
Hospital LOS (> 7 days)	76 (69.1%)	71 (64.5%)	1.11 (0.829–1.485)	0.47
Thromboembolic events:	3(2.7%)	2(1.8%)	1.206 (0.582–2.497)	0.65
Pulmonary embolism (PE)	3 (2.7%)	2 (1.8%)		
Deep vein thrombosis^a^	0	1		
Multiple organ failure	2 (1.8%)	1 (0.9%)		0.50

*TXA* tranexamic acid, *PHTx* prehospital TXA, *H* hospital

^a^One case of deep vein thrombosis complicated with PE (one of the 2 PE cases)

**Fig. 3:**
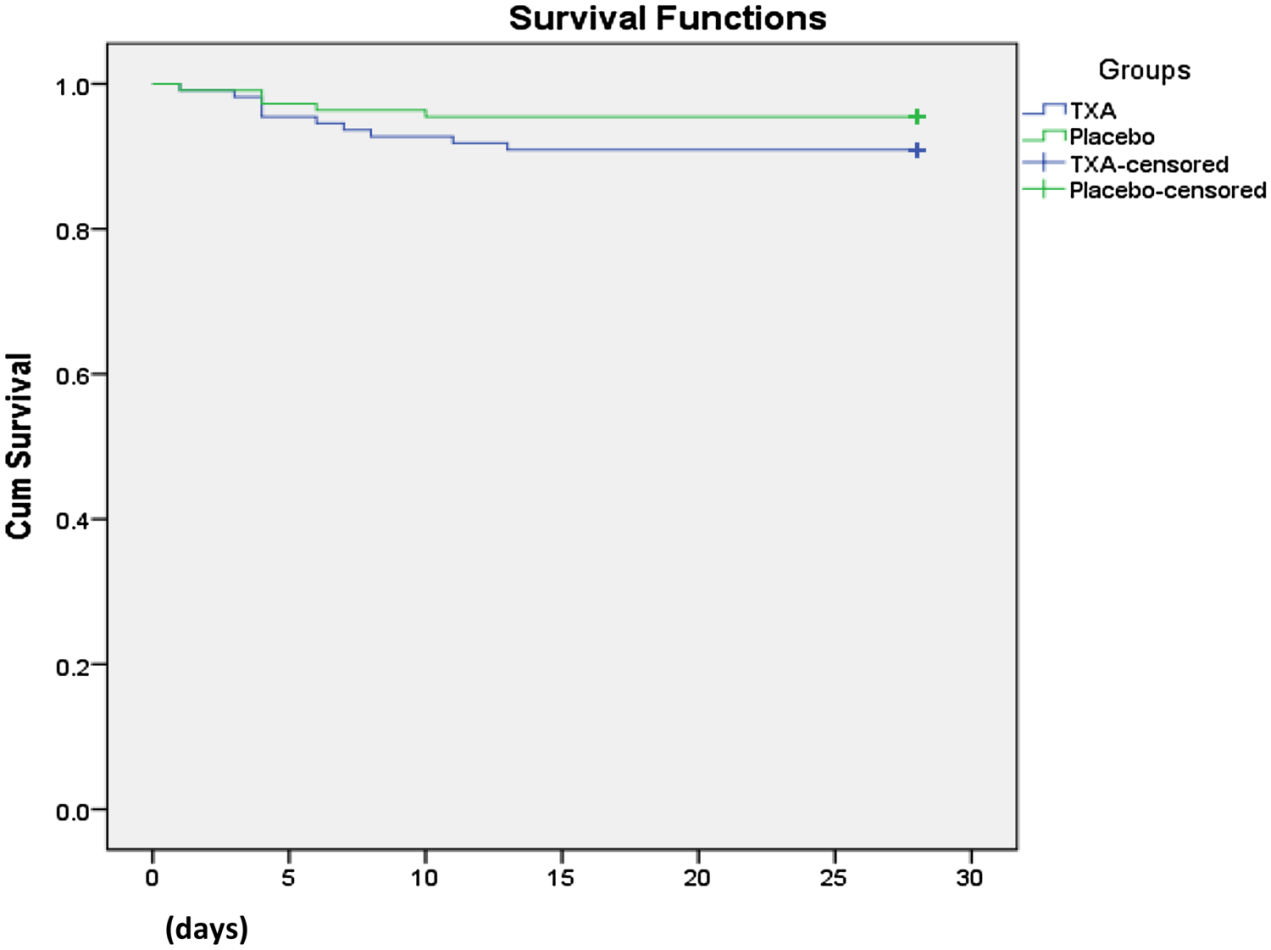
Survival rate 28 days post-injury (Group I TXA vs Group II placebo): log rank (Mantel–Cox) (*p* = 0.18), Breslow (generalized Wilcoxon) (*p* = 0.19), and Tarone–Ware (*p* = 0.19)

Upon comparison of the second dose of TXA to placebo, the placebo arm had non-significantly lower risk of 28-day mortality [OR 0.476 (95% CI 0.157–1.442), *p* = 0.18]. Thus, the second TXA dose had no effect on mortality compared with placebo [OR 1.000 (0.062–16.192); *p* = 0.47]. Kaplan–Meier survival curves showed survival rate at 28-day post-injury for the TXA and placebo groups (log-rank χ^2^ = 1.760, *p* = 0.18) in Fig. 3.

Secondary outcomes: The need of blood transfusions in the first 24 h did not differ significantly between the TXA and placebo groups [OR 1.090 (0.612–1.942), *p* = 0.76] despite the mean number of blood units transfused being higher in the TXA group [5.1 (3.1–7.0) vs. 1.92 (1.14–2.7); *p* = 0.13]. Curiously, the in-hospital TXA group had lower number of MTP activations [OR 2.154 (95% CI 0.579–8.011), *p* = 0.25] than placebo. There were no differences between the two groups regarding hospital length of stay (> 7 days) [OR 0.814 (95% CI 0.464–1.429), *p* = 0.47], multiorgan failure (*p* = 0.50) and thromboembolic events [OR 0.660 (95% 0.108–1.032), *p* = 0.65]. One of the two PE cases in the placebo group also had deep vein thrombosis. There were no documented cases of arterial thromboembolic events including myocardial infarction and stroke during the hospital course.

**Fig. 4:**
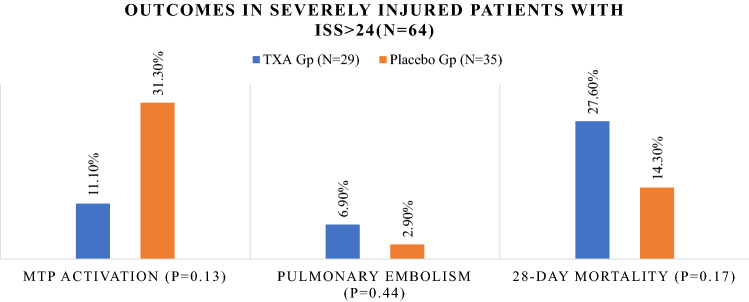
comparison of the outcomes among severely injured patients with injury severity score > 24

Figure 4 shows the outcomes in the two groups among seriously injured patients with ISS > 24 (64 patients: 29 in the TXA group and 35 in the placebo group). In those patients, MTP activation was higher in the placebo group (31.3% vs 11.10%, *p* = 0.13), whereas the pulmonary embolism (6.9% vs 2.9%, *p* = 0.44) and late mortality (27.6% vs 14.3%, *p* = 0.17) were higher in the TXA group but did not reach statistical significance.

## Discussion

To our knowledge, only another single randomized clinical trial [34] has studied the safety and efficacy of a second dose of TXA to injured patients receiving a prehospital TXA dose. The present study demonstrates that while a single early prehospital dose of TXA may be beneficial, a second in-hospital dose may not be required. Thus, the need for a second dose whether liberal, optional, or protocolized remains undetermined.

In 2017, Neeki et al. [44] conducted the Cal-PAT study (multi-centered, prospective, observational cohort study with a retrospective chart review comparison). In that study, the first TXA dose was given prehospital, followed by an optional second dose (neither randomized, nor blind) on arrival at hospital where patients were re-assessed for the need of additional TXA dose. Neeki’s analyses showed no significant differences in mortality or adverse events between the two groups (75 patients prehospital TXA group) compared to pre- and in-hospital TXA group (*n* = 53 patients). However, fewer blood products were transfused to the latter group.

In 2021, a 7-year study on the liberal use of pre- and in-hospital TXA by van Wessem et al. [45] included 422 consecutive trauma patients. The first pre- and in-hospital TXA dose was 1 g bolus, followed by 1 g infusion over 8 h at the discretion of the treating physician. Nearly 80% of all in-hospital TXA doses were given for the suspicion of hemorrhagic shock. Notably, 13% of patients with SBP ≤ 90 mmHg on hospital arrival did not receive TXA at all, while 22% of prehospital TXA and 25% of in-hospital TXA received a second dose based on the SBP alone. The mortality and thromboembolic events were reported in 19% (receiving TXA) and 8% (no TXA), respectively (no significant difference).

Recently, Guyette et al. [34] conducted a double-blind, placebo-controlled, randomized clinical trial in four trauma centers. It assessed the effectiveness and safety of prehospital TXA (1 g) followed by in-hospital administration given in three doses of 0 g (abbreviated group) versus 1 g infusion (standard group) versus 1 g bolus followed by 1 g infusion (repeat group). The authors concluded that prehospital TXA did not result in a higher incidence of thrombotic complications, while TXA did not reduce the 30-day mortality. There was, however, a survival benefit for the subgroup of patients receiving TXA within 1 h of injury and in patients with SBP < 70 mmHg. The limitations of this multicenter trial included the low injury severity (median ISS 11), low blood transfusion requirement (22% in placebo and 17% in TXA group), different prehospital settings and patient management. Moreover, it was underpowered by early termination due to slow enrollment and financial constraints. Compared to our study, late mortality was lower in the standard group (7.8% vs 9.1% in our TXA group) and higher in the abbreviated group (9.3% vs 4.5% in our placebo group). Notably, the lowest mortality in Guyette’s trial was 7.3% for those receiving three TXA doses (repeat group). Unfortunately, the authors did not report the rate of thromboembolism in each TXA dosage group (i.e., 1 vs 2 vs 3 g TXA).

The evidence of benefit to early administration of TXA has led to the growing implementation of prehospital TXA to injured patients at risk of bleeding [1, 7, 12, 13, 18, 46–52]. An earlier retrospective study from our center demonstrated that prehospital TXA administration was associated with a lesser need of in-hospital blood transfusion and MTP activation [35]. Also, TXA did not significantly increase the risk of thromboembolic events or mortality.

The present study demonstrated no significant difference in early and late mortality to administering the second dose of TXA; however, changing the mortality rate in trauma patients is multifactorial. Furthermore, there were no statistically significant differences in blood product administration or duration of hospital stay between the two groups.

A recent retrospective study from Germany [53] compared prehospital TXA (at least one dose) versus no TXA matched group. The study showed no significant difference in thromboembolic events and 30-day mortality, but less massive transfusions for those receiving TXA (5.5% vs 7.2%, *p* = 0.01). Interestingly, 33% of the TXA group and 35% of the control group received TXA on hospital arrival, but the authors did not report the outcomes of those patients.

In the present study and like the other European studies, TBI was the primary cause of death in more than 2/3 of deaths. This finding indicates that TBI may have a bigger role in mortality than hemorrhage, and many of the deaths were unavoidable regardless of TXA. The observation is also consistent with a recent prospective cohort study analyzing prehospital and in-hospital administration of TXA in polytrauma patients [45]. The authors reported that 72% of deaths were attributed to severe head injuries. Lawati et al. [22] demonstrated that TXA did not affect mortality in acute TBI. However, Yokobori et al. [23] analyzed seven RCTs and showed that TXA treatment tended to reduce head trauma-related deaths with no significant incidence of thromboembolic events. The largest RCT on TXA in TBI to date is the CRASH-3 trial [25]. It concluded that TXA treatment within 3 h of injury reduces mild and moderate (but not severe) head injury-related mortality.

### Limitations

This is a single institution randomized clinical trial. Prehospital administration of TXA has some caveats. There is a possibility that SBP remains within normal at the scene or during transportation to the hospital especially in areas with short EMS transportation time and thus precludes the use of TXA; however, the patient could be in the imminent shock stage. It is challenging to identify the potential patients suitable for TXA administration at scene or in ED who sustained severe injuries, but still showed no major signs of deranged physiology [45]. Internal hemorrhage may be difficult to identify, particularly in the prehospital setting, as low blood pressure may have other causes than bleeding and patients in shock grade I and II may initially have normal reading [54]. We did not observe any major side effects of TXA during the prehospital stage, including seizures. The present pragmatic study did not explore the role of TXA based on the patients’ fibrinolysis status and pre-TXA thromboelastography (TEG) was not available. Moore et al. have suggested TXA may worsen or lead to fibrinolysis shutdown and have harmful side effects, which were not explored in this trial [55, 56]. Although prehospital TXA is recommended by Huebner et al., they advised withholding subsequent doses until hyperfibrinolysis is confirmed [46]. However, monitorization of fibrinolysis is not feasible in most trauma centers worldwide and most clinical trials to date have not studied it. There were no clinical signs of traumatic coagulopathy observed in the study groups. The rate of VTE was low as we relied on the clinical manifestation prior to imaging; however, asymptomatic VTE could be overlooked. Screening for DVT/PE was performed at the provider’s discretion. The current clinical trial used prehospital trauma systems in a small country (11,571 km^2^) with a single level I trauma center for an average population of around 2.4 million inhabitants. Consequently, the findings may not be generalizable to other trauma systems and hospitals worldwide. The power calculation was based on the CRASH-2 trial and thus the study was underpowered to draw conclusions. However, the data are supportive that the second dose was unlikely to ever achieve significance, at least as beneficial, but it could reach significance as detrimental in a larger trial.

### Conclusions

The present study found no benefit to use a second 1 g in-hospital dose of TXA to adult injured patients. Although it is multifactorial, the second TXA dose did not change the mortality rate, need for blood transfusion, thromboembolic complications, organ failure and hospital length of stay compared to a single prehospital 1 g dose. Therefore, the common practice of administering a second dose of TXA is questionable and should be revisited. Further larger and multicenter studies are required to establish the ideal dose, frequency, and timing of TXA in trauma patients.
